# Outpatient Oral Doxycycline Therapy for Ocular Syphilis

**DOI:** 10.1001/jamanetworkopen.2024.49364

**Published:** 2024-12-06

**Authors:** Yicheng K. Bao, Jodi Hwang, Christopher Long, Kusha Davar, Nahel Kapadia, Brad Spellberg, Brandon Wong, Narsing Rao, Brian C. Toy

**Affiliations:** 1 Roski Eye Institute, Department of Ophthalmology, Keck School of Medicine, University of Southern California, Los Angeles; 2 Department of Ophthalmology, Los Angeles General Medical Center, Los Angeles, California; 3 Division of Infectious Diseases, Los Angeles General Medical Center, Los Angeles, California

## Abstract

This cohort study compares the outcomes of oral doxycycline therapy with those of intravenous penicillin therapy among patients with ocular syphilis.

## Introduction

The US is experiencing an exponential rise in syphilis incidence, from 6100 cases in 2001 to 203 500 cases in 2022,^1^ with commensurate rises in hospitalizations for syphilitic uveitis.^2^ The US Centers for Disease Control and Prevention–recommended treatment for ocular syphilis, a neurosyphilis equivalent, is intravenous (IV) aqueous crystalline penicillin G 18 million to 24 million units per day for 10 to 14 days. However, IV penicillin has never been established to be superior to potential alternatives in clinical studies. In addition, hospitalizations and indwelling IV lines present significant hazards and costs for the patient and health care system.

Doxycycline has demonstrated clinical and serologic efficacy in the treatment of primary, secondary, early latent, and late latent syphilis.^3,4^ Here, we compare the outcomes of oral doxycycline with IV penicillin therapy for ocular syphilis.

## Methods

We conducted a retrospective cohort study of ocular syphilis diagnosed from January 1, 2017, to December 1, 2023, in Los Angeles. Starting in January 2022, outpatient oral doxycycline (200 mg twice daily for 28 days) was offered as an ocular syphilis treatment through shared decision-making. Patients were identified by electronic medical record (EMR) query. This cohort study was approved by the University of Southern California institutional review board. Informed consent was not required because only deidentified EMR data was collected. This report follows the STROBE reporting guideline.

Nonparametric statistical analyses were used because data were not normally distributed. Categorical variables were compared with the Fisher exact test. Continuous variables were compared with the Kruskal-Wallis test. Data were analyzed with SAS version 9.4 (SAS Institute). Statistical significance was set at *P* < .05, and tests were 2-sided. Data were analyzed from June to September 2024.

## Results

A total of 32 patients with ocular syphilis (median [IQR] age, 46 [36-56] years; 25 males [78%]) were identified and met the Standardization of Uveitis Nomenclature classification criteria for syphilitic uveitis^5^ or had isolated luetic optic neuritis. Sixteen patients (50%) received oral doxycycline (7 patients [44%] received only oral doxycycline; 9 patients [56%] received a short course of parenteral penicillin followed by a full course of oral doxycycline) and 16 patients (50%) received a full course of IV penicillin. There was no difference in the age, sex, race, HIV infection rate, cluster of differentiation 4 (CD4) count, treponemal test positivity, or ocular manifestations between the doxycycline vs penicillin groups (Table 1). The median visual acuity at presentation and at final follow up was better in the doxycycline group than the penicillin group (Table 2). Visual acuity improved or remained stable for 30 of 32 patients (94%). There was no difference in the rates of ocular inflammation resolution among patients who were treated with doxycycline or penicillin. Eleven of 12 patients (92%) were successfully treated with doxycycline without requiring an IV line or hospitalization. A 4-fold decrease in rapid plasma regain (RPR) titers was seen in all patients with at least 9 months of follow up (4 of 4 patients receiving doxycycline [100%]; 7 of 7 patients receiving penicillin [100%]). A sensitivity analysis separating the oral doxycycline only and mixed parenteral penicillin and oral doxycycline subgroups was consistent with the primary analysis results.

**Table 1.** Demographics of Ocular Syphilis Cohort and Treatment Groups

Characteristic	Patients, No. (%)			*P* value
	Complete ocular syphilis cohort (N = 32)	Oral doxycycline (n = 16)	Full IV penicillin (n = 16)	
Demographic information				
Age at presentation, median (IQR)	46 (36-56)	50 (42-57)	41 (34-54)	.52
Sex				
Male	25 (78)	11 (69)	14 (88)	.39
Female	7 (22)	5 (31)	2 (12)	
Race				
Asian	3 (9)	2 (13)	1 (6)	
Black	2 (7)	2 (13)	0	
Hispanic	15 (47)	7 (43)	8 (50)	.58
Non-Hispanic White	9 (28)	3 (18)	6 (38)	
Other^a^	3 (9)	2 (13)	1 (6)	
Baseline characteristics				
HIV positive status				
Yes	17 (53)	7 (44)	10 (63)	.48
No	15 (47)	9 (56)	6 (37)	
CD4 at presentation, medial (IQR), cells/µL	302 (128-332)	233 (35-332)	308 (162-393)	.53
Log HIV viral load at presentation, median (IQR), copies/mL	98 188 (14 907-202 275)	61 094 (12 165-255 670)	104 724 (17 287-161 712)	.59
Treponemal test positive	32 (100)	16 (100)	16 (100)	NA
RPR test positive	32 (100)	16 (100)	16 (100)	NA
CSF VDRL positive, No./total No. (%)	13/18 (72)	3/6 (50)	10/12 (83)	.27

Abbreviations: CD4, cluster of differentiation 4; CSF, cerebrospinal fluid; IV, intravenous; RPR, rapid plasma reagin; VDRL, Venereal Disease Research Laboratory.

^a^ Participants self-reported other race.

**Table 2.** Characteristics of Follow-Up, Uveitis, Visual Acuity, and RPR Titers Compared Between Doxycycline Group and IV Penicillin Group

Characteristic	Patients, No. (%)			*P* value
	Complete ocular syphilis cohort (N = 32)	Oral doxycycline (n = 16)	Full IV penicillin (n = 16)	
Follow-up duration, median (IQR), d	32 (14 to 304)	38 (12 to 255)	24 (14 to 1017)	.91
Follow-up visit at 1 mo (3-6 wk)	20 (63)	11 (69)	9 (56)	.72
Treatment completion verified	27 (84)	14 (88)	13 (81)	>.99
Longitudinal clinical characteristics				
Anatomic category of uveitis				
Anterior uveitis	6 (10)	4 (13)	2 (6)	.15
Intermediate uveitis	2 (3)	0	2 (6)	
Anterior and intermediate uveitis	2 (3)	0	2 (6)	
Posterior uveitis	10 (16)	8 (26)	2 (6)	
Panuveitis	33 (52)	14 (45)	19 (59)	
Isolated optic neuritis	5 (8)	2 (6)	3 (9)	
Resolution of ocular inflammation at 1 mo	15 (75)	9 (82)	6 (67)	.62
Resolution of ocular inflammation at final follow-up	15 (75)	9 (82)	6 (67)	.62
VA at presentation, median (IQR), logMAR	0.57 (0.30 to 1.70)	0.44 (0.18 to 1.09)	1.0 (0.39 to 1.70)	.04
VA at 1 mo after treatment, median (IQR), logMAR	0.40 (0.18 to 0.60)	0.35 (0.10 to 0.60)	0.40 (0.18 to 0.79)	.40
VA at final follow-up, median (IQR), logMAR	0.30 (0.1 to 0.60)	0.18 (0 to 0.51)	0.40 (0.24 to 0.88)	.03
VA change between presentation and final visit, median (IQR), logMAR	−0.11 (−0.76 to 0)	−0.11 (−0.35 to 0)	−0.10 (−0.86 to 0)	.47
RPR titer at presentation, median (IQR)	1:192 (1:64 to 1:512)	1:128 (1:64 to 1:512)	1:256 (1:96 to 1:768)	.48
4-fold Decrease in RPR titer at 6 mo, No./total No. (%)	12/13 (92)	5/6 (83)	7/7 (100)	>.99
4-fold Decrease in RPR titer at 9 mo, No./total No. (%)	11/11 (100)	4/4 (100)	7/7 (100)	NA

Abbreviations: IV, intravenous; logMAR, logarithm of the minimum angle of resolution; NA, not applicable; RPR, rapid plasma regain; VA, visual acuity.

## Discussion

We found that oral doxycycline for the treatment of ocular syphilis may be safe and effective in a selected subset of patients who completed an extended oral antibiotic regimen. Other studies have also demonstrated similar efficacy of oral therapy when compared with IV therapy.^6^ A 4-fold decrease in RPR titers was considered an adequate serologic treatment response and corresponds with resolution of syphilis disease activity. This was observed in all patients with more than 9 months of follow-up. Long-term monitoring is recommended for those treated with doxycycline to ensure clinical and serologic response.

The limitations of this study are its retrospective nature, heterogenous treatment regimens, and lack of longitudinal RPR titers, but this may reflect the clinical setting. Further prospective studies are required to further elucidate the clinical and serologic effects of treating ocular syphilis with oral doxycycline, with the potential benefit to patients of avoiding the harms of prolonged hospitalization and IV catheter or home infusion pump.

## Notes

Supplement.
Data Sharing Statement
