# Supplementary material for: Outpatient Oral Doxycycline Therapy for Ocular Syphilis
Source: JAMA Netw Open. 2024 Dec 6;7(12):e2449364. doi: 10.1001/jamanetworkopen.2024.49364 (PMC11624577; doi:10.1001/jamanetworkopen.2024.49364)
Supplement: Supplement. — Data Sharing Statement [file jamanetwopen-e2449364-s001.pdf]

Data Sharing Statement

Bao. Outpatient Oral Doxycycline Therapy for Ocular Syphilis. *JAMA Netw Open*. Published December 06, 2024. doi:10.1001/jamanetworkopen.2024.49364

Data
Data available: No

Additional Information
Explanation for why data not available: Small cohort study regarding sensitive PHI, risk for re-identification
